# Unraveling the relationship between audience engagement and audiovisual characteristics of automotive green advertising on Chinese TikTok (Douyin)

**DOI:** 10.1371/journal.pone.0299496

**Published:** 2024-04-04

**Authors:** Chuqi Wang, Zhiyu Li

**Affiliations:** Faculty of Humanities and Arts, Macau University of Science and Technology, Macao, China; University of Granada: Universidad de Granada, SPAIN

## Abstract

As video platforms such as Douyin, also known as TikTok’s Chinese version, continue to grow, there is an increasing interest in the study of green advertising videos to understand their audiovisual features and their impact on audience engagement. In this research, we specifically focus on green advertising within the automotive industry. Drawing on literature from sustainability, green advertising, and communication studies, we identified seven audiovisual aspects and three persuasive strategies pertinent to green automotive advertising videos. Utilizing a mixed-methods video analysis framework, we analyzed a dataset of 2,553 green automotive advertising videos on Douyin over three years from 15 June 2020 to 15 June 2023. These videos exhibited higher loudness, a faster pace, and longer durations compared to their non-green counterparts. We categorized three distinct types of green advertising videos on Douyin and established that specific audiovisual features and persuasive strategies are significantly correlated with audience engagement levels. This study not only delineates the audiovisual characteristics of green automotive advertising in China’s digital space but also contributes to the broader discourse on sustainable marketing practices on social networks like TikTok. The findings extend image-centric research to video content and provide marketers with data-driven insights for crafting effective content creation strategies on Douyin.

## Introduction

In recent times, the increased focus on sustainable development and environ-mentally friendly practices has led to a burgeoning interest in green advertising among various stakeholders, including consumers, businesses and academics [1, 2]. At the same time, the meteoric rise in the use of social media by both consumers and businesses has driven the proliferation of green advertising on these digital platforms [3]. Consequently, it is imperative for brands to establish a robust presence within online and social media communications. Kumar et al. [4] note that corporate-generated social media content could potentially influence consumer behaviour.

Despite the significant increase in interest in green advertising and social media in recent years, academic research has predominantly focused on examining the purpose of these articles in terms of information framing and emotional use within green advertising content [3], rather than exploring multimodal content. Previous studies have demonstrated the exceptional impact and persuasiveness of video [5]. At the same time, the undeniable shift from traditional search engines such as Google to emerging plat-forms such as TikTok is capturing consumer attention and increasing market share [3]. Despite this, research on these emerging social media platforms remains relatively scarce.

While the Chinese population is often regarded as one of the least concerned about climate change in the world, the detrimental impact of vehicle emissions on the environment has led to growing public concern [6]. Against this backdrop, many car brands have embarked on green advertising campaigns to promote private car ownership. In this context, the green narratives championed by Chinese automotive advertising are likely to remain a prominent component of the public discourse surrounding the nation’s escalating environmental degradation [7]. Consequently, this study chooses to analyze the accounts of automotive brands on Douyin, a popular social media platform in China.

This manuscript attempts to fill these gaps by exploring the audiovisual features and persuasion techniques used in green advertising videos on Douyin (TikTok’s Chinese counterpart), which has over 600 million daily active users in mainland China [8]. Marketing professionals regard the application as one of the most accessible and efficacious advertising mediums, owing to its rapid expansion and increasingly high levels of engagement [9]. Presently, advertising constitutes the primary commercial model for short video platforms such as Douyin, which serves as a predominant source of revenue [10]. In an effort to augment the efficacy of advertisements, corporations typically employ diverse presentation methods to captivate a broader audience [11]. Therefore, scholarly examination of short video advertising is of paramount importance, as it holds the potential to influence a vast array of prospective consumers [12]. In our study, We examined 2553 videos from ten automotive companies on Douyin, including 160 green advertising videos, based on existing literature on green advertising and audio-visual analysis. We then identified specific audiovisual features and persuasive strategies and classified them into three different types of green advertising videos. We used a mixed effects model to explore the relationship between the characteristics of the short video and the engagement with the video.

Our study seeks to delineate the correlation between green advertising video attributes on Douyin and viewer engagement, and to identify determinants of interaction levels. The insights derived from this research will equip businesses and content creators with refined strategies to enhance audience engagement.

Theoretically, our work expands the literature on green advertising by exploring the influence of audiovisual elements, including graphic and musical components, on viewer engagement—a relatively uncharted aspect to date. Methodologically, we employ a hybrid video analysis framework that integrates automated and manual content analysis, a departure from the predominant reliance on experimental methods or human-based content analysis. Practically, our findings elucidate how audiovisual features and persuasive techniques in Douyin’s green advertisements influence viewer interaction, offering actionable guidance for the production of effective green marketing content.

In the following sections, we first provide an overview of green advertisements on short video platforms and Douyin, followed by a comprehensive discussion of the audiovisual characteristics related to the persuasive strategies of these advertisements, and then propose four research questions accordingly. We then outline our data methodology and findings, and finally consider the implications, limitations, and future directions arising from our findings.

## Literature review

### Video features in green advertising

#### Audiovisual features

Technological advances have gradually changed people’s viewing habits and their preferred devices or platforms for video consumption, positioning videos as a viable alternative to traditional television [13]. Videos encompass multiple modes of presentation, such as text, audio, and visuals, which are processed more quickly and are easier to remember, making them more persuasive than single-text formats [14, 15]. As a result, with the evolution of Internet technology, green video advertising has captured the public’s attention through various social media channels.

We focus on four visual characteristics—brightness, entropy, warm and cool colors.

Advertising has become increasingly dependent on visual aspects [16]. Brightness, as a fundamental color feature at the pixel level, has been shown to be positively correlated with visual working memory (VWM) performance in projects with higher brightness [17]. Furthermore, the relationship between the brightness of fact-checking videos and audience engagement has also been validated [18]. However, it is still un-clear whether the brightness is directly related to the video engagement with the green advertising videos. Higher entropy values indicate greater information complexity [19]. Shen et al. [20] found a positive correlation between the visual complexity of YouTube educational videos and the number of video comments. However, the relationship between entropy and audience engagement in green advertising videos remains unexplored.

Different colors can also affect video engagement. Previous research suggests that viewers’ direct sensory experience of image colors dictates their cognitive responses in persuasion [21]. Studies of green advertising suggest that some green ads use visual elements to evoke environmentally friendly associations, linking the advertised brand to the environment [16]. However, whether these associations elicit user engagement has not been investigated, and it remains unclear whether warm and cool colors influence engagement with green advertising.

In terms of auditory features, we looked at two characteristics: rhythm and loud-ness. Auditory elements can influence people because certain harmonic structures elicit consistent emotional responses [22]. Rhythm, also known as the pace of audio, has been shown to affect emotions, with slow rhythms potentially inducing boredom [23] and faster rhythms reducing negative emotions such as depression and sadness [24]. Loudness significantly predicts audience appreciation after film consumption [25]. Lu et al. [18] found a positive correlation between loudness and video likes and comments in fact-checking videos. As a result, we investigated the rhythm and loudness levels of green advertising videos and their association with the engagement of the viewers.

Video duration is an essential feature of video content. Research has shown that shorter videos tend to be associated with higher levels of viewer engagement [26, 27]. In summary, we formulated our first research question based on the lack of audiovisual research on green advertising videos:

RQ1. What prevalent video features are found in green advertisements on Douyin accounts?

#### Persuasive strategies

In marketing communication, video advertisements often employ specific strategies to intentionally persuade the audience, which can influence audience engagement. Previous studies have shown that emotional appeals in advertisements can induce positive or negative emotions in the audience [28, 29].

First, fear appeals aim to induce fear in the audience by highlighting potential dangers and harms that they may face if they do not adopt the recommended information [30, 31]. Fear appeals are commonly used in political, environmental, and advertising campaigns. In particular, in environmental research, fear appeals can increase people’s awareness of the severity of climate change impacts [32]. Fear appeals also have an impact on people’s environmental attitudes and behavioral intentions [33].

In addition to fear appeals, other persuasive strategies are often used in green video advertising. For example, hope appeals. Hope appeals are emotional appeals that aim to arouse consumers’ hopes and expectations in order to achieve the advertiser’s goals [34]. In a study of climate change advertising, Amy [35] found that hope appeals can have an impact on persuasion outcomes. Research has also found that, under certain conditions, hope appeals in health advertising can be more motivating than fear appeals [36].

Humor appeals are an advertising strategy that persuades people to like a company, brand, product, service or idea by creating laughter and a pleasant feeling [18]. Previous studies have shown that humor strategies can attract audience attention and elicit emotional responses to videos [37, 38]. In addition, humor strategies play an important role in enhancing user engagement with advertising and social media [39]. Based on this, we propose:

RQ2: Are persuasive strategies prevalent in green video advertisements for auto-mobile brands?

RQ3: In green advertising videos, which audiovisual features and persuasive strategies tend to be used concurrently?

### Video features and audience engagement

After delving into the audiovisual features and persuasive strategies of green advertising videos, our aim is to investigate the relationship between these features, persuasive strategies, their combinations and audience engagement. In the current digital landscape, different corporate advertisements on short-form video platforms face fierce competition.

By examining the interplay between video attributes, persuasion tactics and their combinations, we seek to understand the effectiveness of green advertising in capturing audience attention and encouraging participation. In social media, metrics such as views, likes, dislikes, comments, favorites and shares are commonly used indicators of popularity and consumer engagement with videos [40]. These metrics can strongly influence the public perception of videos [41]. These online measurement metrics suggest that audience satisfaction with the content of a post can influence the popularity of a brand’s product or service in the market [42]. Therefore, we propose:

RQ4: Which specific audiovisual features and persuasive strategies in green advertising videos on social media correlate with increased audience engagement metrics, such as likes, comments, favorites, and shares?

## Materials and methods

### Data

Based on the Brand Finance’s top 100 most valuable automotive brands for 2023, we first shortlisted only the brands that had officially verified accounts on Douyin. From this list, we further eliminated brands that did not publish any videos or published fewer than 10 videos. The remaining brands were then pooled together, and a simple random sampling was conducted to select 10 brands. The selected brands were ARCFO Extreme Fox, Polestar Polar Star, TESLA Tesla, Porsche China, Beijing Hyundai, Honda China, SAIC Volkswagen, GAC Group, Haval SUV, and Ideal Auto. Among these, ARCFO Extreme Fox, Polestar Polar Star, and TESLA Tesla are dedicated electric car brands, while Porsche China, Beijing Hyundai, Honda China, SAIC Volkswagen, GAC Group, Haval SUV, and Ideal Auto produce both conventional and electric cars.

We collected all videos from these brands’ account creation until 15 June 2023, covering a period from 15 June 2020 to 15 June 2023. A dataset of 2553 videos and related data (i.e. video titles, video descriptions, and interaction metrics) was obtained. All brand profiles included in this study were established before the date of 15 June 2020, allowing for the collection of video data from the accounts’ inception to the cutoff date of 15 June 2023.

The concept of green advertising is based on three basic principles: advocating a green lifestyle, either with or without the product [43]; reinforcing the environmentally conscious image of the company [44]; and explicitly or implicitly highlighting the relationship between the product and the environment [45]. In this study, green advertising is defined as information that promotes environmentally oriented consumer behaviour [46], promotional messages that can attract consumers who are concerned about environmental protection [47], and information that provides environmental attributes of products or services [48].

#### Dataset information and compliance statement

The dataset used in this study includes a collection of video titles, author usernames, and engagement metrics such as likes, comments, and shares from publicly available profiles on Douyin. All data were collected from public profiles on Douyin, which is a widely used social media platform in China for digital marketing. We ensured that our data collection methods strictly adhered to the terms and conditions of the Douyin platform. The data collected did not contain any personally identifiable information and was following the platform’s privacy policies. Our research team has taken all necessary steps to ensure that the data collection and analysis methods are ethical, non-invasive, and in accordance with relevant data protection laws and guidelines.

### Multimodal automatic analysis framework

#### Audiovisual features

In this study, we selected the main indicators of complexity (entropy), brightness, colour, rhythm, and loudness based on a comprehensive review of audiovisual content analysis literature [18, 49, 50]. These indicators have been shown to have significant relevance in the perception and effects of audiovisual material, providing a robust framework for our multimodal analysis.

For visual feature extraction, we used the OpenCV library to read video files and set an interval of 30 frames for feature extraction [51]. This approach reduces the use of computational resources as the frames contain a large amount of repetitive images [50]. For each selected frame, we calculated its entropy, brightness and colour histogram. Entropy is a measure of texture complexity based on the distribution of pixel values; the shannon_entropy function from the skimage library in Python was used to extract the entropy. Brightness is represented by the average value of the V channel (luminance) after conversion from BGR space to HSV space; colour histogram describes colour distribution information. Finally, the average of all extracted frame features was taken as the visual feature representation of the video. For the auditory feature extraction, we used the MoviePy library to read video files and obtain audio signals. We then converted the audio signals to single channel and used the librosa library to compute rhythm and loudness. Rhythm (tempo) represents the musical beat rate; loudness reflects the intensity level of the audio signal.

The extracted visual and auditory features were stored in an Excel spreadsheet, with one row corresponding to each video file. The columns in the spreadsheet include video title, folder name, entropy, brightness, colour histogram features (512 dimensions in total), rhythm and loudness. Using these methods, we were able to extract visual and auditory features for short videos and store them in an Excel spreadsheet for subsequent analysis. This multimodal automatic analysis method helps to reveal audiovisual patterns in short video content and provides a data base for further research.

#### Persuasive strategies

In green advertising videos, persuasive strategies were coded by two trained coders using a pre-established coding scheme. This scheme involved coding for the presence and intensity of three specific persuasive strategies: Humor Strategy, Fear Appeals, and Hope Appeals. The videos were included in the dataset if they scored at least 1 (rarely) on any of these strategies on a 4-point Likert scale. The inter-coder reliability exceeded 0.75, indicating satisfactory agreement. Any disagreements between the two coders were resolved through discussion until a consensus was reached.

The specific persuasive strategies of Humor, Fear, and Hope appeals were identified and operationalized based on established theoretical frameworks and empirical findings in the field of communication research [18, 35, 52]. Each strategy was included due to its proven efficacy and prevalence in influencing audience reception in prior studies.

- Humor Strategy
  Based on previous research, an advertisement is considered humorous if it contains at least one of the following: puns, jokes, understatement, phrase reversal, double entendres, satire, irony, farce, or incongruity[53]. Coders used a 4-point Likert scale (0 = never, 1 = rarely, 2 = often, 3 = almost always) to annotate the extent to which humor was used in each video.
- Fear Appeals
  Fear appeals are more defined by their effects (i.e., the fear they evoke and the perceived threat) [52]. They primarily include physical fears, social fears, economic fears, and self-esteem fears[54]. Based on these criteria, coders used a 4-point Likert-type scale (0 = never, 1 = rarely, 2 = often, 3 = almost always) to annotate the presence of these fear appeals.
- Hope Appeals
  Hope appeals include two main aspects: evoking hope by offering opportunities and suggesting actions to take advantage of opportunities and achieve expected outcomes[35]. Coders annotated the presence of these appeals to hope using a 4-point Likert scale (0 = never, 1 = rarely, 2 = often, 3 = almost always).

### Analysis

Given the wide range of SD values observed in our dataset, we employed a normalization procedure to reduce variability for variables exhibiting high SD. This transformation was applied to ensure comparability across different scales and to mitigate the potential impact of heteroscedasticity on subsequent analyses.

Descriptive statistics are shown in Table 1. To address RQ1, we computed the raw statistical data for the audiovisual features of all videos posted by the 10 car brands on Douyin in the last three years. Additionally, we compared non-green promotional videos posted by the same car brand accounts as a reference benchmark.

**Table 1:** Audio-visual features, persuasion strategies and video engagement metrics descriptive statistics.

Variable type	Variable name	M	SD
Audiovisual features	entropy	5.91	1.62
	brightness	102.77	50.30
	tempo	122.57	27.38
	loudness	0.15	0.08
	Warm colors rate	0.86	0.15
	Cold colors rate	0.14	0.15
	video length(s)	49.82	69.88
Video Engagement metrics	Video likes	29584.76	105104.77
	Video Comments	636.86	2277.23
	Video Favourites	1364.27	6941.37
	Video Reshares	903.62	2085.04
Persuasive strategies	Fear appeals	0.35	0.69
	Hope Appeals	1.09	0.87
	Humor	0.20	0.59

To address RQ2, we analyzed the average usage rates of audiovisual features within the main persuasive strategies. For RQ3, we computed the correlations between all audiovisual features and persuasive strategies present in the videos in order to identify the coexistence of different audiovisual features or persuasive strategies.

For RQ4, we used a mixed effects linear regression model to predict the associations between video features and video likes, comments, favourites and shares, as well as their relationships with car brand accounts, which served as random effects.

## Results

**Fig 1:**
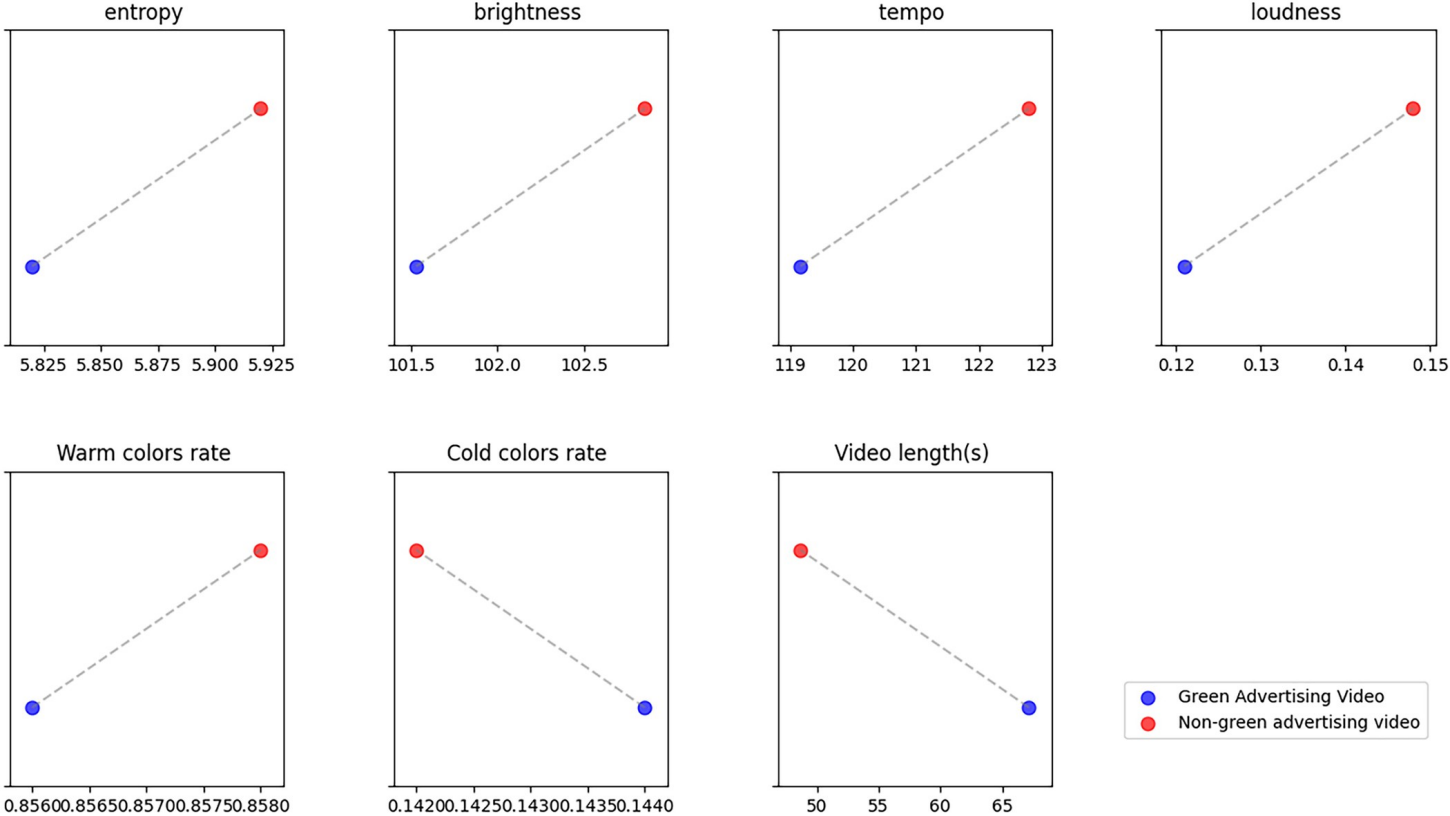
Audio-visual capabilities of green ad videos from the same account as non-green ad videos.

RQ1 investigates the audiovisual characteristics associated with video engagement in green car brand advertisement videos on Douyin. Fig 1 shows the estimated audiovisual characteristics of green advertisements, juxtaposed with non-green advertising videos from the same account. We find that compared to non-green ads on Douyin, green ad videos have significantly higher loudness (M = 0.148), significantly accelerated tempo (M = 122.789), and significantly longer duration (M = 67.181). In contrast, the trends for other attributes are more subtle, making it difficult to draw definitive conclusions.

RQ2 inquires into the persuasive strategies correlated with video engagement. As illustrated in Table 1, among green advertisements of automobile brands on short video platforms, appeals to hope (M = 1.087) are prevalently employed, while fear appeals (M = 0.350) are moderately utilized, and humorous strategies (M = 0.20) are comparatively less frequent.

**Fig 2:**
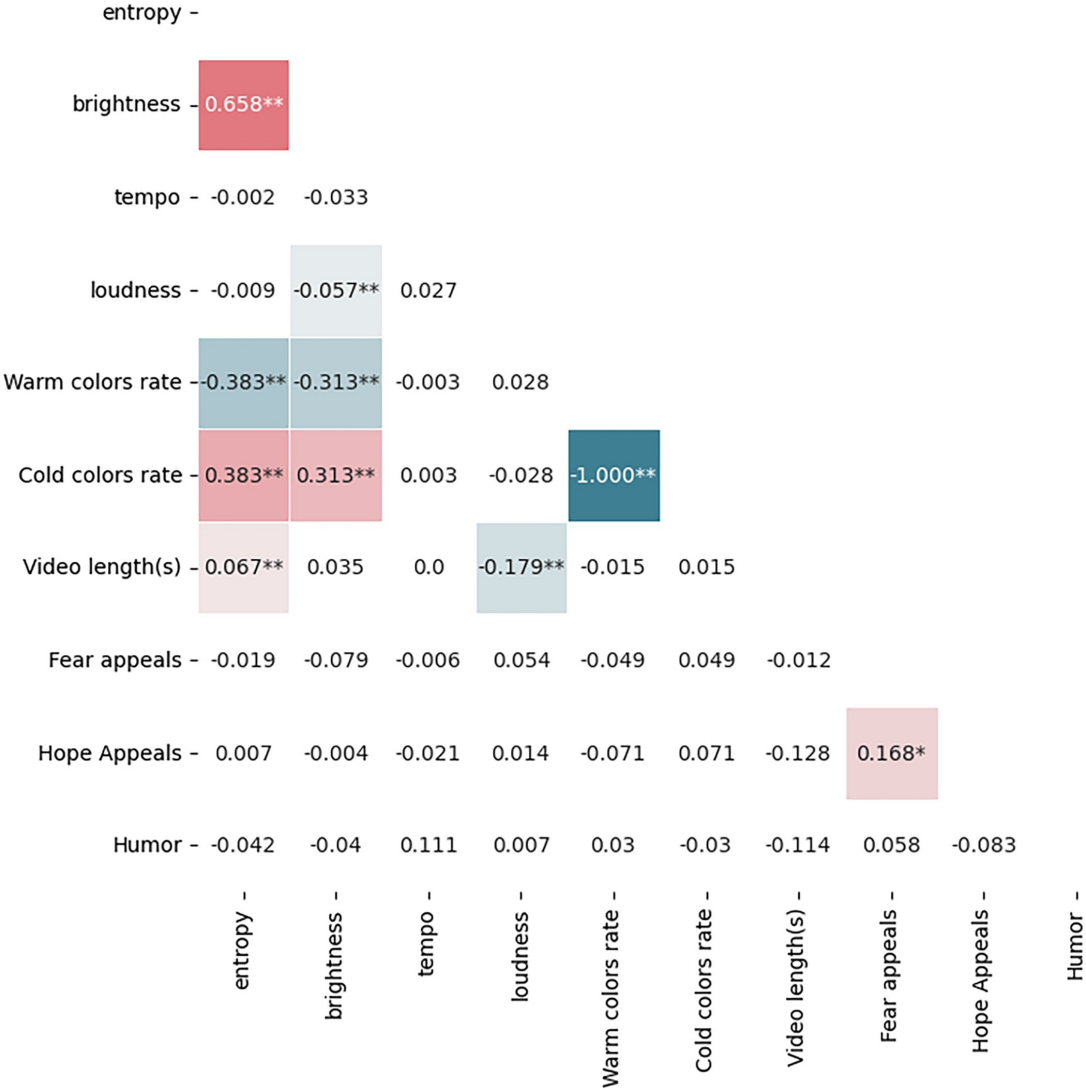
Correlating audiovisual and persuasive features. The table displays the correlation coefficients between pairs of variables. Coefficients within colored areas indicate statistically significant cor-relations at the .01 level.

RQ3 investigates the simultaneous utilization of audiovisual features and persuasive strategies in green advertising on short-form video platforms, as illustrated in Fig 2. Our analysis has revealed several correlations between disparate audiovisual and persuasive components. A robust positive correlation was identified between entropy and brightness (r = 0.658, p<0.01), suggesting a trend for the co-occurrence of complex visual edits with higher levels of brightness in green advertisements. Moreover, a positive correlation between tempo and the incorporation of humor (r = 0.111, p<0.01) was observed, which may reflect a tendency to synchronize humorous content with an upbeat soundtrack. Conversely, there is a negative correlation between the employment of fear appeals and brightness (r = -0.079, p<0.01), indicating that darker visual aesthetics are often used in conjunction with fear appeals in green advertisements. Additionally, a positive relationship exists between the use of fear and hope appeals (r = 0.168, p<0.05), denoting their concurrent deployment in certain green advertisements, potentially to provide a balanced narrative that both highlights the gravity of environmental challenges and inspires positive action.

In accordance with the methodology delineated in the Methods section, three video categories are derived from the cluster analysis (see Table 2).

**Table 2:** Video cluster.

	type		
	Documentary-style	Environmental Action Dissemination	Environmental Day Promotion
Features	M	M	M
Entropy	4.11	6.98	6.64
Brightness	45.69	126.02	133.97
Tempo	121.16	118.44	117.94
Loudness	0.13	0.11	0.12
Warm colors rate	0.92	0.61	0.91
Cold colors rate	0.08	0.39	0.09
video length(s)	79.91	67.61	57.23
Fear appeals	0.34	0.61	0.25
Hope Appeals	1.13	1.29	0.97
Humor	0.18	0.13	0.25

The first type of green advertising videos can be characterised as ’documentary-style’, characterised by long duration (M = 79.911), low brightness (M = 1.90) and appeals to hope (M = 1.13). An example in this category is a video entitled "Joining hands with GQArt and artist Liu Jiayu to explore the origin and pulsation direction of the water of life", published by the Polestar account. In this 2 minute and 28 second video, the creator presents the full story of artist Liu Jiayu driving a Polestar vehicle to follow the language of nature through a short documentary format, calling attention to sustainable development.

Environmental Action Dissemination" is the second type of green promotional video. with moderate duration (M = 67.61), high brightness (M = 126.02), entropy (M = 6.98) and hope appeals (M = 1.29). This category mainly includes green ads related to environmental initiatives of car brands. For example, the video "#Honda is about to launch the fourth phase# of the afforestation project" released by Honda China shows the achievements of Honda’s afforestation efforts in 37 seconds, using bright visuals and diverse content.

The third category of green advertising videos, called "Environmental Day Promotion". These videos have a relatively short duration (M = 57.23) and high brightness (M = 133.97) and typically promote content related to sustainable development events such as World Earth Day and World Animal Day. An example is a 10-second short video released by Audi on World Earth Day, promoting the event through an interactive screenshot game.

In addressing RQ4, we analyzed the impact of specific audiovisual features and persuasive strategies in green advertising videos on social media engagement. Using linear mixed effects models with car brand accounts as random effects (Table 3), we discerned distinct correlations.Our findings reveal no significant relationship between the categorical ’green ad’ type and engagement metrics.

**Table 3:** Multilevel model.

	Variable		Model 1	Model 2	Model 3	Model 4	Model 5	Model 6	Model 7	Model 8
			Video likes	Video Comments	Video Favourites	Video Reshares	Video likes	Video Comments	Video Favourites	Video Reshares
Audiovisual features	entropy						-4830.19	-16.12	-10.54	-46.82
	brightness						100.70	0.53	1.06	-1.13
	tempo						-76.40	0.54	-1.43	0.27
	loudness						45428.55	655.00	1446.02	516.01
	Warm colors rate						46776.21	794.47	-362.89	-743.10
	Cold colors rate						47102.59	453.01	-746.26	-737.41
	Video length(s)						35.15	-0.15	0.29	-0.39
Persuasive strategies	Fear appeals						-1467.70	-1.77	-17.89	-25.95
	Hope Appeals						5767.01	9.18	147.41	56.43
	Humor						-247.30	5.99	13.75	-104.14
	Green Ad Type		-2300.67	35.50	25.59	-75.69	-75.69	-247.30	5.99	13.75
	ICC		0.01	0.26	0.01	0.25	0.03	0.27	0.14	0.26
	Log-likelihood		-1778.79	-1129.56	-1212.60	-1239.29	-1676.69	-1074.57	-1151.16	-1178.18
	Intercept		7049.73	98.44	76.49	410.70	-28489.88	-695.49	298.57	1371.66

Refining the analysis to individual audiovisual features, we observed that video entropy negatively correlates with likes (β = -4830.19, p < .05), suggesting that audiences may prefer less visually complex videos. Brightness exhibited a positive correlation with likes (β = 100.70, p < .05), indicating a preference for visually brighter content. Loudness was positively associated with likes (β = 45428.55, p < .05), comments (β = 325.18, p < .05), and favorites (β = 1446.02, p < .05), signifying that higher auditory intensity resonates more with the audience. Furthermore, the application of hope appeals in persuasive strategies positively influenced likes (β = 5767.01, p < .05) and favorites (β = 147.41, p < .05). These results suggest that optimism in messaging is effective for engaging viewers.

Overall, our analysis suggests that while complex visual structures might disengage viewers, brightness and loudness in videos, alongside hopeful messaging, enhance social media users’ engagement with green advertising content.

## Discussion

With the growing popularity of video sharing platforms in China, green advertising on these platforms requires more attention and research to understand their audiovisual features and how they relate to audience engagement. Drawing on the communication, green advertising and sustainability literatures, we identified seven audiovisual features and three persuasion strategies associated with green advertising videos. Using a combination of automated and manual content analysis, we examined over 2000 short videos posted by car brands on Douyin, a popular short video platform in China. It was found that green advertising videos on Douyin tend to have a higher volume, speed and duration than those of the same car brand that are not green, and often use hopeful persuasion strategies. Considering the interrelated characteristics of the videos, we established three categories of green advertising videos for car brands on Douyin through feature clustering: Documentary Style, Environmental Action Dissemination, and Environmental Day Promotion. Finally, we found that audiovisual features such as brightness, loudness, and pace, as well as hope strategy, are related to audience engagement in green advertising videos. In the following, we will discuss the specific contributions of our study in terms of theory, practice and methodology.

Theoretically, our research provides a significant leap forward in green marketing by systematically identifying and analyzing seven key audiovisual features of green advertisements. By contrasting these with non-green commercials from similar car brands, we offer novel insights that challenge and expand the current understanding of how green marketing operates within the audiovisual domain. Our findings reveal that green advertisements typically showcase increased loudness, tempo, and duration, distinguishing them from conventional advertisements. These unique characteristics contribute to a deeper understanding of green advertising’s visual rhetoric, enriching the academic dialogue about how environmental messages are conveyed and perceived. This work not only enhances the empirical rigor in the study of green advertising but also serves as a foundational reference for future theoretical explorations across various industries.

Our research also advances the academic discourse by establishing a nuanced framework for the analysis of persuasive strategies in green advertisements. We have identified hope, fear, and humor as the principal tactics, with hope appeals being the most predominant in the automotive sector. This finding prompts academic inquiry into the strategic selection and effectiveness of persuasive techniques within environmental messaging. The scarcity of humor and fear appeals observed in our dataset raises questions about their roles and efficacy, which could vary by product category and environmental context. Encouraging further research to probe these dynamics will enable a more sophisticated understanding of how different persuasive strategies influence environmental consciousness and sustainable consumer behavior, thereby enriching the theoretical debate.ns of our study in terms of theory, practice and methodology.

In addition, this study contributes empirical data on the relationship between specific video features and audience engagement in green advertising. Our findings corroborate some aspects of the existing literature; for example, video brightness, loudness, and the use of hope appeals show a positive correlation with at least one engagement metric. Conversely, entropy exhibits a negative correlation with engagement metrics. However, results for other characteristics, such as video color bias, video duration, humor appeals, and fear appeals, diverge from previous studies. This discrepancy may stem from our research methodology—a mixed content analysis approach that may not isolate the impact of each individual feature while controlling for others. Indeed, many features interact with one another. For instance, while some research suggests a positive correlation between video length and audience engagement, we did not observe this effect. This difference could potentially be explained by the divergence in video themes—fact-checking content versus green car advertising—with longer videos being preferred for fact-checking but not as favored for advertising. These insights underscore the necessity of visual content analysis for green advertising videos, the imperative to explore videos of varying themes, and the need for rigorous experimental research. Our findings provide guidance for advertisers and content creators on short video platforms to maximize their impact, helping them make evidence-based decisions on content creation tailored to engagement metrics.

From a methodological standpoint, our study offers a significant contribution by refining the mixed video analysis framework. This advancement moves beyond the prevalent human-centric content analysis methods and allows for a more comprehensive, efficient evaluation of green advertising content. By tailoring specific indicators to better suit the analysis of green advertisements, we have set a new methodological benchmark for the field. This innovative approach facilitates a more robust and systematic exploration of audiovisual and persuasive features in green marketing research, which can be applied across different sectors to enhance the validity and reliability of future studies.

This investigation is subject to five primary limitations. The first constraint arises from the dataset’s composition, which encompasses a modest selection of video advertisements from ten automotive brands featured exclusively on the social media platform Douyin. Consequently, the generalizability of our findings may be limited. Secondly, the heterogeneity of video quality, which is contingent on user-controlled settings in non-WiFi environments on Douyin, was not accounted for within our experimental design. This factor could introduce variability in user engagement metrics, potentially confounding the analysis. Thirdly, there is an omission of a limitation here which should address the third point. A fourth limitation pertains to the operationalization of persuasive strategies; the tripartite criteria of hope, fear, and humor may not encapsulate the full spectrum of persuasive techniques employed within green advertisements, possibly skewing the interpretative framework. Finally, the accuracy of video selection and the application of indicators may be compromised by the proprietary algorithms of Douyin and the overarching influence of internet censorship in China, necessitating a cautious approach to the extrapolation of these results.

Future research directions should pivot towards four promising avenues. The application of humor in green advertising, particularly within the automotive sector, emerges as a potentially fruitful area of further investigation, given its underrepresentation in the current sample. Expanding the corpus of green advertising videos to scrutinize the nexus between humorous content and audience engagement would be a valuable endeavor. Additionally, implementing experimental methodologies to isolate and quantify the impact of video quality on user engagement would enhance our understanding of its influence. Further inquiries might also benefit from considering the effect of industry-specific contexts and backgrounds on the efficacy of green advertising strategies. Finally, adopting multimodal analytical techniques to compare the characteristics of short-form social media videos with green advertising content on traditional video platforms and television could provide comprehensive insights into the effectiveness of environmental messaging across various media landscapes.
